# Bioimpedance Patterns and Bioelectrical Impedance Vector Analysis (BIVA) of Body Builders

**DOI:** 10.3390/nu15071606

**Published:** 2023-03-25

**Authors:** Cristian Petri, Matteo Levi Micheli, Pascal Izzicupo, Niccolò Timperanza, Tommaso Lastrucci, Daniele Vanni, Massimo Gulisano, Gabriele Mascherini

**Affiliations:** 1 Department of Sport and Informatics, Section of Physical Education and Sport, Pablo de Olavide University, 41013 Sevilla, Spain; cpet2@alu.upo.es; 2 Exercise Science Laboratory Applied to Medicine “Mario Marella”, Department of Experimental and Clinical Medicine, University of Florence, 50134 Florence, Italy; matteo.levimicheli@unifi.it (M.L.M.); niccolo.timperanza@stud.unifi.it (N.T.); tommaso.lastrucci@stud.unifi.it (T.L.); danielevanni88@gmail.com (D.V.); massimo.gulisano@unifi.it (M.G.); gabriele.mascherini@unifi.it (G.M.); 3 Department of Medicine and Aging Sciences, University “G. D’Annunzio” of Chieti-Pescara, Via L. Polacchi, 11, 66100 Chieti, Italy

**Keywords:** body composition, bodybuilding, muscle mass, muscle hypertrophy

## Abstract

Bodybuilders are athletes characterized by high muscle mass. During competitions, the evaluation is performed based on aesthetic parameters. The study aims to provide normative references of body composition with the vector bioimpedance methodology (BIVA). A second aim is to compare BIVA assessments performed on both sides and the upper and lower body. A group of 68 elite bodybuilders (41 males aged 30.1 ± 9.2 years and 27 females aged 32.1 ± 8.0 years) was enrolled. A BIVA assessment was performed the day before the 2021 World Natural Bodybuilding Federation Italian Championships. As a result, male and female bodybuilders ranked to the left in the BIVA ellipse relative to the general population. Furthermore, unlike females, males also ranked lower than the general athletic population. In addition, in the symmetry assessment, males show a significantly greater upper body than the lower, right, and left parts, while in women, this is observed for the lower part of the body. The differences in the results obtained between males and females can be attributed to the different patterns of endocrine production between the sexes and the different criteria used by the juries to attribute the final score during the competitions. Therefore, BIVA references in bodybuilders could help adjust the training and nutritional program during the peak week before a competition.

## 1. Introduction

Body composition assessment is currently a part of functional assessment in sportspeople, allowing the monitoring of athletes’ nutritional and health status and checking the state of physical fitness. The study of the relationship between body composition and sports performance is a growing research area in sports science. In particular, some sports, such as gymnastics and running, have a gravitational component; therefore, reduced fat mass values can favor success in competitions. Alternatively, many combat sports are weight-classified; therefore, athletes must stay within a specific range of body mass. Finally, sports characterized by expressions of strength and/or speed, such as sprints in athletics or weightlifting, benefit from the presence of large muscle masses. However, in some competitions, success is attributed based on the aesthetic characteristics of the participants. In this context, monitoring body composition has become fundamental, and in aesthetic sports can provide additional and helpful information [1].

Bodybuilding is an aesthetic sport, and a prerequisite for a successful bodybuilder is to have the expected muscular morphology [2]. Specifically, bodybuilding is a combined aesthetic and weight-class sport in which physical appearance, muscular mass, symmetry, shape, and definition are the evaluation parameters with specific references based on the category in which each athlete competes [3]. Therefore, the goal of a bodybuilder is to attain maximal body muscle by focusing on hypertrophy training and, in preparation for a competition, striving for excellent muscular definition [4].

Among the methods developed to assess body composition, bioelectrical impedance analysis (BIA) has long been an easy and viable technique for obtaining qualitative and quantitative assessment in bodybuilders. However, the studies predominantly used the conventional BIA evaluation, which studies and describes the different body tissues (mainly fat-free and fat-mass) [5]. This approach has some limitations, including sensitivity to hydration status, potential inaccuracy of predictive equation when applied to populations with different characteristics, and a minimum standard error of estimation, even when the equation is accurate [6,7].

An innovative use of BIA consists of evaluating the raw bioelectrical parameters, such as resistance (R), reactance (Xc), and phase angle (PhA) through bioelectrical impedance vector analysis (BIVA) that allows the evaluation of the vector position compared with population-specific tolerance ellipses [8]. Furthermore, studying bioelectrical properties in athletes made it possible to create specific references helpful in monitoring body composition during the competitive season [9]. Recently, studies have investigated the positioning within the ellipse of the R-Xc graph of specific sports populations within the athlete’s population, in particular, the bioelectrical characteristics of soccer players [10], cyclists [11], handball players [12], synchronized swimmers [13], and rowers [14] have been studied. Furthermore, side-by-side differences in bioelectrical parameters have been found to be more pronounced in tennis players than in the general population, suggesting this method can be useful to identify body asymmetries in those sports with unilateral loading [15].

The study through the BIVA assessment of bodybuilders could increase the knowledge of the body composition of these athletes, providing specific positioning within the R-Xc graph, given the high muscularity associated with low levels of fat mass that characterize this particular study population. This study hypothesizes that bodybuilders have a position far to the left within the R-Xc graph, with a high phase angle (PhA). Therefore, this study aims to perform a BIVA assessment on a sample of bodybuilders to provide the population-specific tolerance ellipses. A secondary aim of this study is to compare BIVA assessments performed on both sides and on the upper and lower body (hand to hand and foot to foot, respectively). We hypothesize that male and female bodybuilders will show different BIVA patterns according to the sexual dimorphism in body masses distribution.

## 2. Materials and Methods

### 2.1. Participants

During the Italian championships 2021 of the World Natural Bodybuilding Federation, athletes were required to participate voluntarily in the study. The inclusion criteria were (1) to be a member of the World Natural Bodybuilding Federation Italy, (2) to be over 18 years old, (3) to have finished at least sixth in the final ranking, (4) to not use drugs that can induce muscle hypertrophy, and (5) be of white ethnicity.

A group of 64 elite bodybuilders (37 males aged 30.1 ± 9.2 yrs with body mass index = 24.5 ± 1.5 kg/m^2^ and 27 females aged 32.1 ± 8.0 yrs with body mass index = 19.8 ± 1.3 kg/m^2^) was enrolled in the study after receiving written informed consent. The categories in which the male participated were: eleven subjects in Classic Physique, sixteen in Man Physique, and fourteen in Bodybuilding. At the same time, eight females participated in the Figure and nineteen in the Bikini. The study was carried out in conformity with the ethical standards in the 1975 Declaration of Helsinki.

### 2.2. Procedures

The recruitment and evaluation of the participants were conducted on the morning of the day before the competition; therefore, during the peak week [2], the athletes showed their optimal body composition (i.e., the lowest fat mass and the highest muscle mass). Weight was measured to the nearest 0.1 kg and height to 0.5 cm (Seca GmbH & Co., Hamburg, Germany). Body mass index (BMI) was then calculated as weight divided by height^2^ (W/H, expressed as kg/m^2^).

Whole-body BIVA is the body tissues’ opposition to the flow of an electric current. It is the vector sum of the resistance (R, Ω)—the major resistance to the current through intracellular and extracellular ionic fluids—and the reactance (Xc, Ω)—the additional opposition due to the capacitive elements such as cell membranes, tissue interfaces, and non-ionic substances. The raw bioimpedance parameters R, Xc, and Phase Angle (PhA, calculated as the arc tangent of Xc/R × 180°/π) were obtained using a phase-sensitive segmental bioelectrical analyzer (BIA 101 BIVA PRO, Akern, Florence, Italy) with a current of 250 μA at a single frequency of 50 kHz. It was calibrated every morning using a calibration circuit procedure of known impedance (R = 380 Ohm, XC = 47 Ohm, 1% error) supplied by the manufacturer. After cleaning the skin with isotropy alcohol, four low intrinsic impedance adhesive electrodes (Biatrodes Akern Srl, Florence, Italy) were placed on the hand’s back and the other four electrodes on the neck of the corresponding feet, respecting the standard protocol [16]. The proximal hand electrode was between the radial and ulnar styloid processes, and the distal hand electrode was positioned in the center of the third proximal phalanx. The proximal foot electrode was placed directly between the medial and lateral malleoli at the ankle, and the distal foot electrode was proximal to the second and third metatarsophalangeal joints. Therefore, through this procedure, with a single impedance measurement, it was possible to collect all the data to evaluate both the whole body and the body side evaluation.

The whole body BIVA assessment uses R and Xc parameters, standardized for the subject height to remove the effect of conductor length, producing a vector plotted in an R-Xc graph. Vector length indicates the hydration status from fluid overload (reduced R, short vector) to dehydration (increased R, longer vector), and lateral migration of the vector due to low or high Xc indicates a decrease or increase in mass dielectric (membranes and tissue interfaces) of soft tissues. The individual vector can be ranked on the R-Xc point graph against tolerance ellipses representing 50%, 75%, and 95%, according to the values of a reference population [17].

The body sides BIVA assessment was performed on the right side (hand to foot right side), left side (hand to foot left side), upper side (hand to hand), and lower side (foot to foot) of the body with the subjects in a supine position with their arms and legs abducted. The evaluation parameters were:

- resistance/height and reactance/height for the upper body (R/hup and Xc/hup);
- resistance/height and reactance/height for the upper body (R/hlo and Xc/hlo);
- resistance/height and reactance/height for the right side of the body (R/hrt and Xc/hrt);
- resistance/height and reactance/height for the left side of the body(R/hlt and Xc/hlt) [14].

In order to verify muscle mass through raw bioelectrical values, the Levi Muscle Index, defined as LMI = (PhA × H)/R, was calculated [18]. BIA measurements were all taken by the same-trained investigator to avoid inter-observer errors.

### 2.3. Statistical Analysis

The normality of the data was verified by applying the Shapiro–Wilk test and descriptive statistics were calculated for each independent variable, as reported in Table 1. The bioelectrical impedance variables followed the Gaussian distribution. Differences between the BIVA modalities for the raw bioelectrical parameters were evaluated through the analysis of variance with repeated measures (RM-ANOVA) on the within-subject factor (right side, left side, upper side, and lower side). The Bonferroni–Holm method was used for the post hoc test, and the sphericity was corrected using the Greenhouse–Geisser method when the condition of equal variance was violated. Each participant was plotted in the tolerance ellipses (50%, 75%, and 95%) of the Italian reference population of the same sex [19]. A two-sample Hotelling’s T^2^ test was used to determine the BIA vector differences concerning the reference population [19] and the athletic population [20] and for BIVA modalities (right and left side, upper and lower body) comparisons. The Mahalanobis’ test calculated distances between ellipses [21]. A *p*-value < 0.05 was considered significant. IBM SPSS 23.0 (SPSS, Chicago, IL, USA) was used for statistical calculations, and BIVA software [21] was used for plotting and comparing the bioelectrical parameters, as well as for computing the tolerance ellipses (50%, 75%, and 95%) of the investigated sample.

## 3. Results

Table 1 describes the general and anthropometric characteristics of the sample for both sexes.

**Figure 1:**
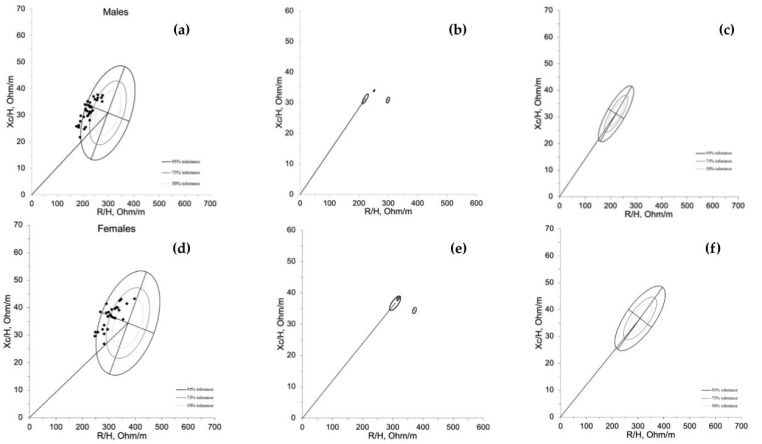
Whole-body BIVA graph of male and female natural bodybuilders. Caption: (**a**) Whole-body BIVA point graph of the male bodybuilders plotted on the tolerance ellipses of the athletic reference. (**b**) Whole-body BIVA mean graph for the whole-body mean impedance vectors of the male bodybuilders and the athletic and general reference population. (**c**) Specific 50%, 75%, and 95% tolerance ellipses of male natural body builders. (**d**) Whole-body BIVA point graph of the female bodybuilders plotted on the tolerance ellipses of the athletic reference. (**e**) Whole-body BIVA mean graph for the whole-body mean impedance vectors of the female bodybuilders and the athletic and general reference population. (**f**) Specific 50%, 75%, and 95% tolerance ellipses of female natural body builders.

The BIVA point graph indicates that male natural bodybuilders mainly fell outside the 75% percentile (Figure 1a). Furthermore, most bodybuilders fell in the lower left quadrant area to the left of the impedance vector, as previously indicated by Campa for the athletic population [20]. Male bodybuilders were statistically different compared with the general male population [19] and athletic reference [20]. Specifically, they fell more to the left than the general population (T^2^ < 168.8; D = 2.14; *p* < 0.001) and to the left and down than the athletic population (T^2^ < 17.7; D = 0.95; *p* < 0.001) (Figure 1b) and their specific the 50%, 75%, and 95% tolerance ellipses are represented in Figure 1c. On the other hand, female bodybuilders compared with the general female population [18] and athletic reference [20] fell to the left of the general population (T^2^ < 67.3; D = 1.64; *p* < 0.001) but did not differ from the athletic population (T^2^ < 1.9; D = 0.27; *p* < 0.40) (Figure 1d,e). The specific 50%, 75%, and 95% tolerance ellipses of female natural bodybuilders are represented in Figure 1f. The LMI value of male bodybuilders is 3.7 ± 0.6, while for female bodybuilders, it is 2.3 ± 0.4.

**Figure 2:**
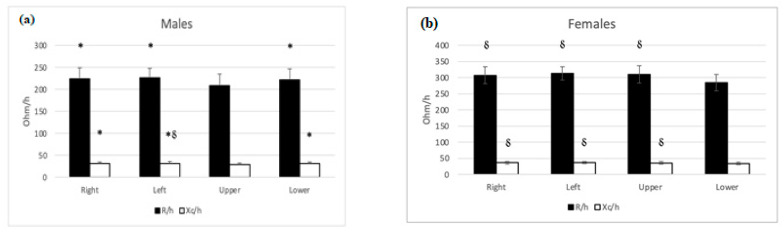
Bioelectrical characteristics of the sample for both sexes, for right and left sides and upper and lower body. (**a**) males. (**b**) females. caption: R/h = resistance/height; Xc/h = reactance/height. * Significantly different from the corresponding upper body bioelectrical parameter; § Significantly different from the corresponding lower body bioelectrical parameter.

Bioelectrical characteristics of both sexes, for the right and left sides and the upper and lower body, are shown in Figure 2. Significant differences for both sexes were found for R/h (males: *F*(1.3, 45.4) = 18.0, *p* < 0.001, *partial eta squared* = 0.33; females: *F*(1.3, 34.2) = 30.6, *p* < 0.001, *partial eta squared* = 0.54) and Xc/h (males: *F*(1.2, 43.2) = 30.8, *p* < 0.001, *partial eta squared* = 0.461; females: *F*(1.3, 32.8) = 10.5, *p* < 0.001, *partial eta squared* = 0.29). Post hoc analysis showed that in males, the R/h of the upper body significantly differed from the R/h of the lower body (*p* < 0.02), right (*p* < 0.001), and left (*p* < 0.001) sides, respectively. Similarly, the Xc/h of the upper body significantly differed from the Xc/h of the lower body (*p* < 0.01), right (*p* < 0.001), and left (*p* < 0.001) sides, respectively. Furthermore, the Xc/h of the lower body was significantly different from the left side (*p* < 0.005) (Figure 2a). On the other hand, in females, the R/h of the lower body significantly differed from the R/h of the upper body (*p* < 0.001), right (*p* < 0.001), and left (*p* < 0.001) sides, respectively. In comparison, the Xc/h of the lower body significantly differed from the Xc/h of the upper body (*p* < 0.004), right (*p* < 0.001), and left (*p* < 0.001) sides, respectively (Figure 2b). No differences were found in PA for both sexes.

**Figure 3:**
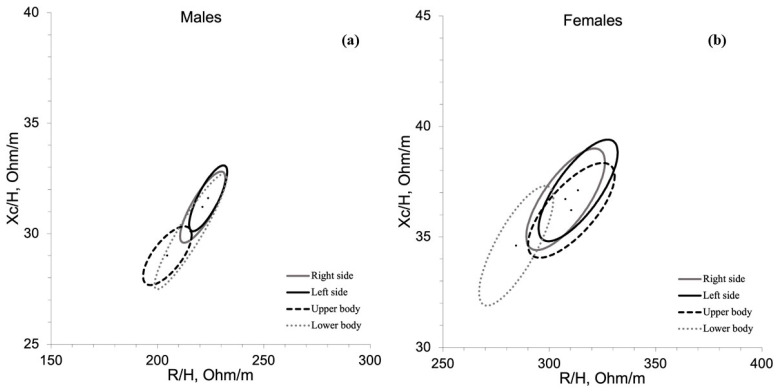
Bioelectrical characteristics of the sample for both sexes, for right and left sides and upper and lower body. Caption: (**a**) BIVA vectors of the right and left sides and on the upper and lower body in males. (**b**) BIVA vectors of the right and left sides and on the upper and lower body in females.

Figure 3a compares the BIVA vectors assessed on the right and left sides and on the upper and lower body in males. The Hotelling’s T^2^ test indicates that the upper body bioelectrical vector was significantly shorter than the corresponding vectors assessed on the right (T^2^= 76.6; F = 38.8; D = 1.44; *p* < 0.001) and left (T^2^ = 76.6; F = 38.8; D = 1.44; *p* < 0.001) sides. Figure 3b compares the BIVA vectors assessed on the right and left sides and on the upper and lower body in females. The Hotelling’s T^2^ test indicates that the lower body bioelectrical vector was significantly shorter than the corresponding vectors assessed on the right (T^2^= 76.6; F = 38.8; D = 1.44; *p* < 0.001) and left (T^2^ = 76.6; F = 38.8; D = 1.44; *p* < 0.001) sides, and upper body (T^2^ = 76.6; F = 38.8; D = 1.44; *p* < 0.001).

## 4. Discussion

This study has two aims: (1) to provide for the first time the population-specific tolerance ellipses for BIVA assessment in bodybuilders, and (2) to compare bioelectrical parameters of the upper, lower, right, and left sides.

The hypothesis of the first aim has been verified, as the body structure, endowed with high muscularity, places the study sample in the left quadrant of the ellipse. In detail, male bodybuilders differ from the general population and athletes in being more left-leaning [19] and, therefore, with higher PhA. However, they also rank lower in BIVA ellipses than athletes: they take a higher water content to increase muscle mass due to higher hydration status. The results of the female bodybuilders report differences compared to the general population that is superimposable to the male counterpart [19]. On the other hand, no differences are described concerning the population of athletes. The sample of female athletes described by Campa et al. [20] showed a higher body mass index than the female bodybuilders of this study. Therefore, it is possible to speculate about the search for a slimmer shape by female bodybuilders, probably to have advantages in the judging phase based on the aesthetic character of the competition.

Generally, in peak week, bodybuilders increase aerobic exercise in their training program [22] and re-modulate the carbohydrate loading to increase muscle glycogen storage [23,24] due to the imminence of competition. In particular, a reduction in carbohydrates from 5.3 to 3.8 g/kg/day has been described in the transition from the non-competitive to the competitive phase [25]. However, reducing carbohydrates of this magnitude close to the competition should only happen briefly to avoid muscle loss [26]. Furthermore, approaching the competitive phase, a 5–6% reduction in body fat mass is sought; therefore, the total energy intake is reduced, and proteins and fats are modulated [27]. Increasing the protein content during diets with energy deficit regimes allows the maintenance of muscle mass. Therefore, on average, bodybuilders close to competition are advised to maintain protein values up to 3.1 g/kg of lean body weight during severe calorie restriction [28]. Fat intake is generally the lowest of the three macronutrients and, like carbohydrates, reduces over time in favor of protein maintenance [29]. The optimal strategy of combining training and nutrition for the peak week has yet to be established [29]. Peak week goals are to maximize the volume of muscle mass with the most defined aspect. Therefore, maintaining resistance training levels and increasing protein intake is an established procedure. However, the increase in aerobic exercise [27] for greater muscle definition may lead to a depletion of muscle glycogen with a consequent reduction in the volume of muscle masses. At the same time, the reduction in carbohydrate intake could have the same effect as the reduction in muscle volume due to the reduced fluid recall exerted by the lower muscle glycogen content [24].

The study’s second aim was to analyze the different BIVA patterns based on the upper, lower, right, and left body sides. Currently, there are few studies targeting BIVA by body segments. Unfortunately, this paucity in the literature may not allow an appropriate comparison with the results of the present study because the other studies used tools with different work frequencies [30] or did not show all vector impedance parameters [31]. A recent study indicates that BIA discriminated more pronounced asymmetries of bioelectrical parameters in tennis players than in the general population, suggesting this method can be useful in assessing the body characteristics of athletes competing in sports with a dominance of unilateral loading [15]. Thus, the authors believe that evaluating the single body side could be a new direction of study in an aesthetic discipline where symmetry and proportion are reported as a parameter of judgment for the final score between the right and left and between the upper and lower sides. The results of the present study show that the upper body is more relevant in males (Figure 3a). In contrast, the lower body is more relevant for female bodybuilders (Figure 3b): this could be due to the greater muscle mass expressed in different body parts between the sexes. In addition, male and female hormone action differences play a decisive role in final body composition outcomes [32].

The study of body composition using bioimpedance recently has a new orientation toward the vector evaluation of raw bioelectrical parameters: this has promoted the possibility of independent evaluation from formulas in which sex, age, weight, and ethnicity are parameters of these predictive equations [6]. Furthermore, the elaborations of the reference ellipses for different sporting and non-sporting populations were followed. In this context, providing bodybuilders’ BIVA references for the first time will be helpful for (1) comparison with athletes from other sports given the muscularity that characterizes this specific sports population; (2) providing training feedback to bodybuilders themselves; therefore, they can adjust the combination of nutrition and training to reach their optimal level. In this context, the evaluation of muscle mass using the Levi Muscle Index (LMI), a new parameter based on raw bioelectrical parameters, is also useful. In detail, the male bodybuilders in this study show higher values than male elite soccer players (3.7 ± 0.6 vs. 3.08 ± 0.35, respectively) [18].

Some studies [14,33,34] link bioelectrical parameters, mainly phase angle [35], with sports performance. However, the physical performance of bodybuilders would not be directly related to the probability of victory, as the aesthetic nature characterizes bodybuilding. Therefore, morphological aspects influence the final score more than higher strength values.

Currently, the bioimpedance assessment performed on some specific body areas does not have a single denomination. Instead, there are simultaneously terms such as “regional,” “segmental,” and “district,” which in some cases are used as synonyms, but each has its specific meaning. Therefore, greater homogeneity in the terminology of this specific area is advisable to ensure correct scientific progress in this field of research.

Future directions of study could be:

1. increase the sample size of bodybuilders in the BIVA study, which would make it possible to provide both normative reference values for this specific population and a left border of the ellipse in the R-Xc graph of the sports population that are more reliable from a statistical point of view.

2. Deepen the evaluation of body sides in sports where symmetry is a parameter that can determine success in a competition. This could be done by increasing the number of bodybuilders evaluated and by carrying out symmetry assessments on both large body areas (e.g., upper–lower or right–left sides) and small body areas (e.g., right–left tight or right–left arm) and then using the localized impedance measurement technique.

### Strengths and Limitations of the Study

This study has strengths. The first is the sample size, which aligns with the studies on larger numbers of bodybuilders. Second, the enrolled athletes participated in the national championship and were all enrolled as competitive athletes, unlike some studies of amateur athletes. Thirdly, it is the first study to perform total bioelectrical and body side assessment on bodybuilders.

However, this research is not without its limitations. First, the subjects are from the same country; therefore, the results may only be generalizable to some athletes worldwide with different ethnicity. Secondly, the muscle mass values recorded in the present study’s sample may be lower than in other studies (the BMI value falls within the normal weight range). However, the authors have chosen this federation to avoid drug use that could compromise the reliability of the physiological hypertrophic response. Third, enrolled athletes fall into different categories, following different metrics that could influence training goals; however, this heterogeneity could also provide a broader picture of possible responses to bodybuilding practice.

## 5. Conclusions

In summary, this study provided, for the first time, the BIVA ellipses for the athletic bodybuilder population. In addition, the high muscularity that characterizes these athletes allows us to provide the hypothetical left margin of the BIVA ellipse of the general athletic population and therefore have a reference.

Furthermore, this study highlights asymmetries between the upper and lower body in both sexes. This aspect could compromise the competition result, as the symmetry, intended not only between the right and left sides, is part of the judgment parameters for the final score.

## Figures and Tables

**Table 1:** General and anthropometric characteristics of the sample for both sexes.

Variable	Mean ± SD	Min–Max	Mean ± SD	Min–Max
Age (years)	30.1 ± 9.2	19.0–54.0	32.1 ± 8.0	20.0–48.0
Body mass (kg)	72.8 ± 6.8	59.8–90.6	53.7 ± 5.5	40.7–63.0
Height (cm)	172.2 ± 5.9	162.8–190.0	164.6 ± 7.0	149.0–179.0
Body mass index (km/m^2^)	24.5 ± 1.5	21.2–29.2	19.8 ± 1.3	17.9–22.6

## Data Availability

Data can be obtained from Gabriele Mascherini on a reasonable request gabriele.mascherini@unifi.it.
